# The Bioconversion of Sewage Sludge to Bio-Fuel: The Environmental and Economic Benefits

**DOI:** 10.3390/ma12152417

**Published:** 2019-07-29

**Authors:** Adam Smoliński, Janusz Karwot, Jan Bondaruk, Andrzej Bąk

**Affiliations:** 1 Central Mining Institute, Pl. Gwarków 1, 40-166 Katowice, Poland; 2 Sewage and Water Supply Ltd. Rybnik, Pod Lasem str. 62, 44-210 Rybnik, Poland; 3 Institute of Chemistry, University of Silesia, Szkolna 9, 40 007 Katowice, Poland

**Keywords:** thermal utilization, new materials for energy industry, biowaste, toxic waste, biofuel, co-combustion

## Abstract

This paper aims to analyze the economic feasibility of generating a novel, innovative biofuel—bioenergy—obtained from deposit bio-components by means of a pilot installation of sewage sludge bio-conversion. Fuel produced from sewage sludge biomass bears the potential of being considered a renewable energy source. In the present study, 23 bioconversion cycles were conducted taking into consideration the different contents, types of high carbohydrate additives, moisture content of the mixture as well as the shape of the bed elements. The biofuel was produced using post fermentation sewage sludge for industrial energy and heat generation. Based on the presented research it was concluded that the composite biofuel can be co-combusted with hard coal with the optimal percentage share within the range of 20–30% w/w. Sewage sludge stabilized by means of anaerobic digestion carried out in closed fermentation chambers is the final product. The average values of the CO_2_, CO, NO, NO_x_ and SO_2_ concentrations in flue gas from co-combustion of a bioconversion product (20% w/w) and coal were 5.43%, 1903 ppm, 300 ppm, 303 ppm and 179 ppm, respectively. In total, within a period of 4.5 years of the plant operation, 1853 Mg of fuel was produced and successfully co-combusted with coal in a power plant. The research demonstrated that in the waste water treatment sector there exists energy potential in terms of calorific value which translates into tangible benefits both in the context of energy generation as well as environmental protection. Over 700,000 Mg of bio-sewage sludge is generated annually in Poland. According to findings of the study presented in the paper, the proposed solution could give 970,000 Mg of dry mass of biomass qualified as energy biomass replacing fossil fuels.

## 1. Introduction

The development of sewerage infrastructure and household sewage connections has become a priority objective in addressing the issue of preventing soil and water pollution in Poland. Households, along with other sources of sewage, are connected to sewerage systems which route the effluents to a waste water treatment facility in order to remove the contaminants. Sewage sludge is a by-product generated in the process of industrial and municipal waste water treatment. However, with the development of sewage infrastructure, the volume of sewage sludge increases and, as a consequence, there arises the problem of its proper management.

Sewage sludge is a dispersive system in which the non-dispersive phase is a liquid phase in the form of water with dissolved substances while the dispersed phase constitutes a solid phase in the form of insoluble parts or a gaseous phase in the form of a gas dissolved in liquid [1]. Sewage sludge bears certain potential of practical application on account of the fact that it contains organic substances as well as biogenic elements [2,3]. The volume of organic substance in dry mass ranges from 2.6% to 11% in hydrated sludge whereas in the dehydrated one up to more than 50%. Therefore, the sludge chemical content makes it suitable to be reused as a soil fertilizer. Yet, due to the presence of heavy metals and pathogenic microorganisms, there arises a need to comply with legal standards determining the requirements to be met in order to use sewage sludge in agriculture (this applies only in the case of untreated sewage sludge) [4,5].

Currently, sewage sludge can be utilized also as a soil improver or for engineering purposes in degraded land reclamation after determining the permissible level of heavy metals concentrations in compliance with the regulation of the Minister of Environment on municipal sewage sludge [6]. Moreover, sewage sludge with suitable mixtures can be used for phytoremediation purposes. If there is no possibility of agricultural utilization of sewage sludge, or if such a mode is legally restricted, sewage sludge is subject to landfill or thermal disposal [7,8,9,10,11,12]. Both the above methods increase the costs of wastewater collection services connected with the treatment process. In addition, the legal requirements resulting from Poland’s membership of the European Union (EU) have a considerable impact on the available methods of sewage sludge management. For example, the increased legal requirements led to the ban of sewage sludge landfill disposal from 1 January 2016 and, as a result, to the growing volume of generated sewage sludge [4,13]. The problem of the most effective and law-abiding processing of sewage sludge affects all waste water treatment facilities, including the one in Orzepowice, Poland. After the processes of dehydration and sanitation, the sewage sludge leaves the technological line and requires further handling; its annual volume amounts to approximately 7 thousand Mg.

In light of biding legal regulations, the landfilling of sewage sludge is the least desirable method of its management. Due to its changeable content, the sludge generated during the process of wastewater treatment constitutes a major problem in terms of its stabilization and utilization. So far, there does not exist a single optimal method of sewage sludge management. The selection of the technology is made on an individual basis and depends on the size of the particular wastewater treatment facility as well as on the characteristic of the processed sewage [14].

A construction of an incineration plant on the premises of the wastewater treatment facility in Rybnik Orzepowice, Poland, would be a capital-intensive project. Besides, the combustion process produces ash of a high concentration of heavy metals which is considered hazardous waste [15].

In addition, the incineration process is accompanied by heat generation for which there is no demand on the premises of the wastewater treatment facility. It is biogas combustion that provides the heat necessary for technological purposes, central heating and hot water. Excess heat would have to be released to the atmosphere.

The activities aiming at reducing the volume of landfilled sewage sludge and increasing the degree of sewage sludge conversion as well as the development of thermal conversion technologies are in accordance with the preferred European standards and legal regulations [16].

Fuel produced from sewage sludge biomass bears the potential of being considered a renewable energy source (RES) [17,18,19,20]. In parallel, it is a fuel which does not have an adverse impact on biodiversity or on the available arable land resources and a product which also solves the issue of sewage sludge landfilling.

The aim of this paper is to analyze the economic feasibility of biofuel production using an innovative technology of sewage sludge management which may constitute an alternative to thermal utilization of sewage sludge.

## 2. Materials and Methods

In light of the existing limitations concerning natural management of sewage sludge which result from legal regulations and the scarcity of potential areas suitable for alternative utilization, thermal disposal acquires more and more importance. The thermal methods of sludge stabilization and utilization include incineration, co-incineration as well as pyrolysis [17,18,19]. Our activities regarding the bioconversion of sewage sludge correspond with the trend. A pilot installation of sewage sludge bioconversion was built at the waste water treatment facility in Orzepowice, Poland.

### 2.1. Materials

The research was focused on the development and implementation of a cost-effective environmentally safe technology of waste recycling and utilization, including energy recovery, by means of thermal and biochemical conversion processes [21,22]. In the period between May 2012 and December 2016, the total amount of 1853 Mg of biofuel was produced and provided as an innovative fuel to a power plant for co-combustion with coal in electricity generation. The pilot installation of sewage sludge bioconversion was designed to achieve the production capacity of approximately 4500 Mg of biofuel per year. The designed capacity of the prototype installation allows 60% reduction of the currently generated volume of sewage sludge. The installation enables an ongoing in situ processing of sewage sludge as well as its utilization as a qualified biomass in the energy sector. The process is fully pressurized, and to a large extent, it reduces the environmental impact of the sewage plant by the elimination of landfilling and the necessity of transporting the sludge to remote traditional utilization sites. The installation is characterized by the application of sewage sludge bioconversion consisting in the generation of biofuel in the process of stimulated maturation of the mixture composed of sewage sludge, high carbohydrate content biofuel as well as balanced amounts of reactive components and activated cultures of bacteria. During the process of maturation, microbiological oxidation of the mixture occurs, which leads to the conversion of microbes consisting in the cracking of their biochemical structure with the generation of biologically neutral decomposition elements.

The physical and chemical parameters which characterized the stabilized sewage sludge are presented in Table 1. The analyses of physical and chemical parameters of fundamental importance for characterization of stabilized sewage sludge were performed in the accredited laboratory of the Center for Environmental Research and Natural Hazards (Ledziny, Poland). These were made with the application of the relevant standards, testing procedures and the following apparatuses and techniques: potentiometrically (pH acc. to PN-EN 12176:2004), gravimetric analysis (content of dry mass and organic matter acc. to PN-EN 12880:2004 and PN-EN 12879:2004, respectively), ICP-OES (content of P, Ca, Mg, Cd, Cu, Ni, Pb, Zn and Cr acc. to PB-114/08.2013), spectrophotometry (content of ammonium nitrogen and total N acc. to PB-091/02.2012) and atomic absorption spectrometer (content of Hg acc. to PB-076/10.2012). Shaping the post conversion biomass into moldings and their subsequent thermo-inclusion allow for secure transportation and utilization in the form of biofuel. Additionally, moldings with sintered surface have a moist core releasing water vapor, which decreasing the temperature of the CO to CO_2_ combustion process and, in consequence, the emission of dioxins and NO_x_.

### 2.2. Method

**Figure 1:**
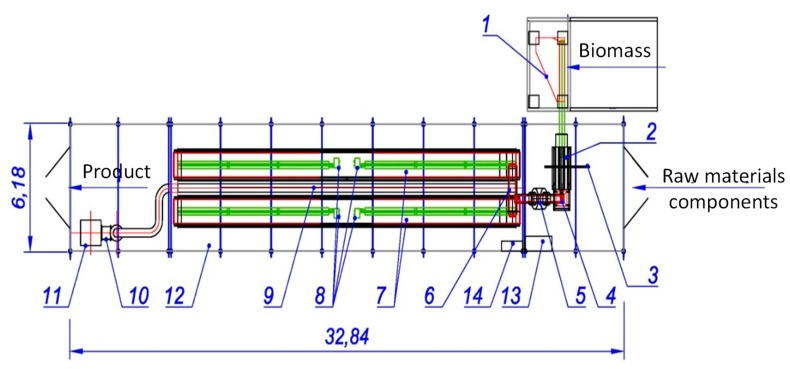
The bioconversion installation for sewage sludge conversion to bio-fuel: 1. Container for post-fermentation biomass; 2. Extruder of formed biomass; 3. Electro-actuation of components; 4. Feeder; 5. Microwave device; 6. Reverse conveyor; 7. Reticular conveyors; 8. Tubular radiator; 9. Extraction ventilation channel; 10. Duct fan; 11. Column of conditioning and hygienization of post-conversion gases; 12. Biomass bioconversion reactor; 13. and 14. Control system.

The bioconversion installation processes sewage sludge to produce a surrogate to be used for the purpose of professional energy generation (see Figure 1). The obtained product—biofuel—can be easily and safely transported and utilized as a biofuel in industrial energy plants or in heating plants. It can be also used in municipal waste incineration facilities increasing the calorific value of the feedstock. The bioconversion installation consists of a homogenizer and a sewage sludge bio-converter where the bioconversion process takes place. The biomass produced in the bio-converter is routed to a shaping device and then in the form of moldings to a thermal stabilizer where it is subject to the thermo-inclusion process. The thermal stabilizer is powered by hot exhaust gases from natural gas combustion. The exhaust gases leaving the thermo-inclusion chamber are streamed through a granular filter for the purpose of de-dusting; next, they are directed to a thermostatic node in order to heat the process air which is streamed to the bio-converter. The gases produced during the bioconversion process are routed from the bio-converter to the purification node where they are purified and then blended with the exhaust gases from the thermo thermo-inclusion process. The exhaust gases are routed to a thermal hygienization node in a torch equipped with a catalyzer and a concentric system to channel wet gases with a parallel removal of the condensate. Post fermentation biomass of 80% hydration degree constitutes the feedstock whereas the final product is biofuel with the heat of combustion ranging from 16.6 to 18.9 MJ/kg and 30% moisture content.

Within the research framework, 23 bioconversion cycles were conducted taking into consideration the different contents, the types of high carbohydrate additives, moisture content of the mixture as well as the shape of the bed elements. The research was conducted for an optimal content of the mixture presented in Table 2.

## 3. Results and Discussion

The analyses of bioconversion, stabilization (thermal inclusion), combustion and emissions confirmed that a sphere of 20 mm diameter constitutes an optimal shape and size of the surrogate. Moldings of such shape and size characterize of optimum porosity of the bed within the bioconversion and stabilization processes, good mechanical resistance, uniform combustion and the size of the molding fitting into the pea coal category, which in the case of co-combustion with hard coal has a beneficial effect on the bed structure.

Based on the research findings, it was observed that the composite biofuel can be co-combusted with hard coal while the optimal percentage share is within the range of 20–30%. Table 3 presents the average values of the concentration of emissions released during the combustion of different fuels.

The obtained results contributed to the development of a new generation, composite biofuel technology dedicated for industrial energy generation and district heating, including various forms of energy co-generation. Sewage sludge stabilized by means of anaerobic digestion carried out in closed fermentation chambers is the final product. The organic substance in the municipal waste water is an element of food chain; therefore, it must be classified as biomass of zero CO_2_ emission and absorption in the process of photosynthesis and combustion. As distinct from simple forms of biomass, the only difference is the location of the said biomass in the food chain.

As a result of anaerobic digestion of waste water the following three streams are produced:

1. Biogas mainly composed of methane, carbon dioxide, hydrogen sulfide and water vapor used directly for the purpose of heating or energy co-generation;
2. Post fermentation biomass which after dewatering forms sewage sludge categorized as waste;
3. Filtrate rerouted to the preliminary node of waste water purification.

Streams 1 and 3 are not classified as waste. Stream 2 which does not have a direct application is classified as waste according to traditional technology of waste water treatment. The obtained research results enabled to redefine the categorization of the sewage sludge into “post fermentation biomass” which in the innovative waste water treatment technology is utilized as a component of a composite biofuel constituting a final fuel product dedicated for the energy and energy co-generation sectors. The sequential bioconversion of post fermentation biomass allowed to yield a market value composite biofuel.

### 3.1. Environmental Benefits

The primary objective of the research was the production of a fuel suitable for green energy generation by means of energy biomass combustion in industrial power plants and heating plants. Equally important was the issue of complying with the requirements stipulated in the EU Directive 2009/28/EC of the European Parliament and of the Council of 23 April 2009 on the promotion of the use of energy from renewable sources.

Nationwide implementation of the method enabled the production of approximately 830–950 thousand Mg of dry energy biomass with calorific value in the range of 14–18 GJ/Mg. The implementation of the technology significantly improved the available schemes of obtaining plant biomass for energy purposes and at the same time mitigated the sequestrated fuel balance in Poland. Additionally, the novel product, as an alternative to traditional plant biofuels, has tremendous environmental implications because it replaces monoculture farming and contributes to sustaining forest resources which are scarce in Poland. Other important benefits include the improvement of local ecosystems in the context of sewage sludge management, the elimination of interim repository sites characterizing of odor nuisance as well as the necessity of hygienization. Research projects which aim at improving the thermal methods of sewage sludge management bear the potential of decreasing its volumes discharged to the environment. The fact that the above activities also result in producing electricity and heat, reducing in this way the combustion of conventional energy carriers, for example hard coal, creates a synergy effect which can be translated into tangible economic as well as environmental profits [23].

### 3.2. Profitability Analysis

The profitability analysis of the product encompasses cost savings resulting from decreasing the expenditures connected with hygienization and transportation of sewage sludge and the income from selling the final product of bioconversion as well as the costs of energy biomass production. The analysis is based on the assumption that the final product will be contracted by a major industrial electricity and/or heat generation plant. The form of the fuel allows long distance transportation; however, it has an impact on the price.

#### The Calculation of Operational Costs

In regard to the calculation of operational costs, it was assumed that 5475 Mg/year of sewage sludge characterized by 80% moisture content is placed in the homogenizer together with a total volume of 471 Mg/year of deposit biomass, activated bacteria cultures as well as reactive components. The ingredients are next mixed in the homogenizer; the obtained mixture of 5946 Mg/year is routed to the bio-converter where the process of an effective maceration takes place. After the bioconversion process has been completed, the biomass is subject to shaping and thermo inclusion to achieve the final energy biomass in the volume of 2245 Mg/year. Table 4 presents the operational costs and mark-ups.

Table 5 presents the calculation of the profitability of sewage sludge based production of energy biomass. The profitability analysis demonstrated that the sale of the final bioconversion product bears the potential of reducing the costs of waste water treatment by 141,490 EUR.

Within the framework of the development phase, risk assessment and sensitivity analyses were carried out against the changes of market and economic conditions concerning the sale of the bioconversion product. A reassessment demonstrated that the variability of market parameters falls within the predetermined range, which proves the economic viability of the project. The conducted analyses also confirm the beneficial environmental impact due to eliminating the need of repository and transportation. Another important aspect is the compliance with the requirements of the EU Directive of reducing CO_2_ emissions through the production of energy biomass dedicated for professional electricity and heat generation.

## 4. Conclusions

1. The method may be applied in any waste water treatment facility with a modest capital expenditure. Energy biomass constitutes the final product to be used in the energy sector obliged to co-firing of biomass with fossil fuels in accordance with the EU policy.
2. The volume of bio-sewage sludge currently generated in Poland converted to dry mass accounts for over 700,000 Mg. Using the proposed method, it is possible to produce, on the national level, biomass in the amount of 970,000 Mg of dry mass qualified as energy biomass replacing fossil fuels and directly dedicated for professional electricity and heat generation.
3. At the same time, the processed sewage sludge is not landfilled. The fact that renewable resources of sewage sludge biomass are utilized for energy purposes instead of exploiting crop and forest cultivations may be considered as yet another advantage. The product is not a competitor in relation to agricultural food production.
4. The research demonstrated that in the waste water treatment sector there exists energy potential in terms of calorific value which translates into tangible benefits both in the context of environmental protection and professional energy generation.
5. The conducted economic analysis confirmed that the sewage sludge bioconversion product may constitute an alternative biofuel to achieve the optimal composition of the required energy mix, and most importantly, a product which does not increase the unit cost of producing 1 MWh of electrical energy.

## Figures and Tables

**Table 1:** Physical and chemical parameters of the stabilized sewage sludge.

No	Parameter	Unit	Mean Value
1	pH	-	10.85
2	Dry mass	%	23.13
3	Organic matter content	% dry mass	45.50
4	P content	% dry mass	1.78
5	Ca content	% dry mass	14.81
6	Mg content	% dry mass	0.55
7	ammonium nitrogen content	% dry mass	0.33
8	Total N content	% dry mass	5.05
9	Cd	mg/kg d.m.	1.06
10	Cu	mg/kg d.m.	179.17
11	Ni	mg/kg d.m.	10.93
12	Pb	mg/kg d.m.	18.83
13	Zn	mg/kg d.m.	504.83
14	Hg	mg/kg d.m.	0.49
15	Cr	mg/kg d.m.	25.70

**Table 2:** Optimal content of the mixture.

Ingredient	Share
Post-fermentation biomass—stabilized municipal sewage sludge 19 08 05	70%
Additive 1: Flotoconcentrate—flotation concentrate of hard coal	10–20%
Additive 2: Cellulose lignin	0–10%
Additive 3: Wood sawdust	0–5%
Additive 4: Glycerol bio-phase from the rapeseed oil transesterification process	4–5%
Additive 5: A mixture of bioalcohols as a co-product of alcoholic fermentation	1%

**Table 3:** Average values of the concentration of emissions during different fuel combustion.

Fuel	Emission Concentrations					
	O_2_ %	CO_2_ %	CO ppm	NO ppm	NO_x_ ppm	SO_2_ ppm
Coal	13.12	6.56	1558	163	162	249
Coal + 20% of bioconversion product	14.44	5.43	1903	300	303	179

**Table 4:** Operational costs and mark-up (EUR/Mg of the product).

Specification	Value
Depreciation	10.38
Materials	11.06
Energy	10.85
Salaries	9.41
Real property tax	0.59
Equipment costs (repository sites)	3.84
Maintenance	1.06
Direct cost of 1 Mg of product	47.18
Overhead costs mark-up	4.24
Overall costs mark-up	5.65
Margin	5.65
TOTAL	62.71

**Table 5:** Profitability of sewage sludge based production of energy biomass.

Specification	Value (EUR)	EUR/1 Mg of the Final Product
1. Transportation savings 5475 Mg (sewage sludge feedstock) 23.53 EUR/Mg	128,832	57.37
2. Income from the sale of bioconversion product 2245 Mg (production)	140,784	62.71
3. Production operational costs	128,126	57.06
TOTAL (1 + 2 − 3)	141,490	63.02
